# Antibacterial Activity of Eight Medicinal Plants from the Traditional Pharmacopoeia of Niger

**DOI:** 10.1155/2023/6120255

**Published:** 2023-07-24

**Authors:** Mahamane Idi Issa Abdoulahi, Melogmo Dongmo Yanick Kevin, Tchokouaha Yamthe Lauve Rachel, Hama Hamadou Habibou, Bakasso Sahabi, Alio Sanda Abdelkader, Fabrice Fekam Boyom, Ilagouma Amadou Tidjani

**Affiliations:** ^1^Laboratory of Natural Substances and Organic Synthesis, FAST, Abdou Moumouni University, BP 10662, Niamey, Niger; ^2^Laboratory for Phytobiochemistry and Medicinal Plants Studies, University of Yaoundé I, P.O. Box 812, Yaoundé, Cameroon; ^3^Pharmacology Laboratory, Institute of Medical Research and Medicinal Plant Studies (IMPM), B.P. 6163, Yaounde, Cameroon; ^4^Laboratory for Management and Valorization of Biodiversity in Sahel, Faculty of Science and Technology, Abdou Moumouni University, BP 10662 Niamey, Niger

## Abstract

The emergence of multidrug bacterial resistance poses a great public health problem and requires a constant search for new antibacterial agents. However, Niger's flora possesses several medicinal plants used in traditional medicine to cure infectious diseases and can be used as sources of bioactive ingredients. This current study was designed to evaluate the antibacterial activity of eight plants used in the traditional pharmacopeia of Niger. The extracts were prepared by maceration using ethanol, methanol, and distilled water. The obtained extracts were screened against *Salmonella* spp., *Shigella* spp., and *Escherichia coli* using the microdilution method coupled with a resazurin-based assay. Phytochemical screening was performed using colorimetry, while the quantification of total polyphenols, total flavonoids, and total tannins was determined by spectrophotometry. Out of the eight plants obtained, five named *Cassia italica*, *Limeum pterocarpum*, *Phyllanthus pentandrus*, *Strychnos innocua*, and *Ximenia americanum* exhibited antibacterial activity with MICs ranging from 500 *μ*g/mL to 2000 *μ*g/mL. Phytochemical screening showed the presence of alkaloids, saponosides, tannins, flavonoids, terpenes/sterols, quinones, and polyphenols. The ethanolic and methanolic extracts of *X. americana* contained important quantities of total polyphenols, with 43.59 ± 0.15 and 41.97 ± 0.02 mg EAG/100 mg of extract, respectively. These extracts showed the highest contents of total tannins at 46.49 g/L and 45.52 g/L, respectively. For total flavonoids, the highest content was obtained with the methanolic extract of *P. pentandrus*, with 3.12 ± 0.01 mg QE/100 mg of extract. These findings justify the uses of these plants in traditional medicine for the treatment of infectious diseases such as diarrhea and can be used as starting points for the development of phytodrugs against infectious diarrhea.

## 1. Introduction

Infectious diseases are pathologies caused by microorganisms such as fungi, viruses, and bacteria. The latter is responsible for most of the pathology, including diarrhea. Diarrhea is mainly caused by bacteria and viruses. Since the vaccine against rotavirus was approved, bacteria have become the leading cause of diarrhea [1] and are classified as the second most common cause of death in children under five years of age, with approximately 525,000 deaths each year [2]. *Shigella* spp., *Salmonella* spp., and *E. coli* are the principal bacteria responsible for diarrhea. In Niger, *Shigella* is the leading etiology of diarrhea, with 60.5% of episodes of severe shigellosis occurring mainly in children under 5 years of age [3]. Globally, the overwhelming majority of cases have occurred in developing countries, where poor sanitation and limited access to clean water facilitate the spread of enteric pathogens. Malnutrition and lack of proper care are major contributors to the high mortality rate, particularly among young children [4]. The treatment of diarrhea is mainly based on the administration of antibiotics. For this reason, the WHO recommends the use of azithromycin and antibiotics from the fluoroquinolone family, particularly ciprofloxacin, as the first line of therapy [5]. Unfortunately, the emergence of multidrug-resistant bacterial strains and the failure of this treatment led the WHO to include these bacteria on the list of 12 priority bacteria for which the search for new drugs is urgently needed [6]. Regarding the history of traditional medicine, interest in herbal medicines continues to increase. Indeed, many patients are increasingly practicing herbal self-medication [7]. The flora of Niger is still rich and unexplored, and exploring these plants based on traditional uses can be the best avenue to search for new drugs against infectious diarrhea and to provide the population with effective new drugs without harmful effects and at a low cost. These results will make it possible to obtain scientific data that validate the uses of these plants in traditional medicine. The objective of this research is to evaluate the antibacterial activities of some medicinal plants of the traditional pharmacopoeia of Niger.

## 2. Materials and Methods

### 2.1. Plant Material and Extract Preparation

Using information gathered from traditional uses, some plants were selected. Different plant parts from each selected plant were harvested in September 2020 in Kollo (Tillabery). Small specimens were taken for the department of biology, and their scientific names were provided by the department of biology by comparison to the specimen deposit under the number of herbariums. Once in the laboratory, the plant parts were washed to remove all dirt, cut, and dried at room temperature for two weeks. After that, the plants were ground into a fine powder and kept for further study. Traditionally, the solvent most commonly used is water. In order to justify scientifically the activity of these plants, an aqueous extract was performed for each plant. Organic extracts were chosen to see the effect of the extraction solvent on the activity. The extracts were prepared by maceration using two organic solvents (ethanol and methanol) and distilled water. For this, 100 g of powder was soaked in 1000 mL of each solvent. The solutions were stirred twice each day for 3 days. At the end of this period, the extracts were filtered using hydrophilic cotton, and the filtrates were concentrated at 40°C under pressure of 175 mbar for the ethanolic extract and 337 mbar for the methanolic extract using rotavapor (Heidolph). The crude extracts were dried under ventilation at room temperature to remove the remaining solvent and kept at 4°C for further studies.

### 2.2. Biological Material

Five clinical isolates, *Salmonella enterica typhimurium* CPC, *Salmonella enterica typhi* CPC, *Salmonella enterica enteridis* CPC, *Shigella dysenteria* CPC, and *Escherichia coli* CPC, from the *Centre Pasteur du Cameroun* and three reference strains, *Shigella flexneri* NR518, *Shigella sonnei* NR519, and *Escherichia coli* ATCC25922, provided by BEI Resources to the Antimicrobial and Biocontrol Unit, University of Yaounde 1, Cameroon, were used in this study. These bacteria were kept at 4°C and revived 24 hrs prior to each assay on Muller-Hinton agar (Sigma-Aldrich) at 37°C.

### 2.3. Antibacterial Activities of Crude Extracts

#### 2.3.1. Preparation of Stock Solutions of Extracts and Reference Antibacterials

Stock solutions of extracts were prepared at 100 mg/mL by dissolving 100 mg of extracts in 1 mL of 10% DMSO. Amoxicillin (Sigma-Aldrich) used as a positive control was prepared under the same conditions at 1 mg/mL by dissolving 1 mg of powder in 1 mL of acidified distilled water.

#### 2.3.2. Preparation of Bacterial Suspension

The different bacterial suspensions were prepared according to the 0.5 McFarland standard. For this, a stock suspension was prepared at turbidity 0.5 McFarland standard (corresponding to an approximate concentration of 1.5 × 10^8^ CFU/mL) from 24-hour cultures on Muller-Hinton agar (MHA) and then diluted to 10^6^ CFU/mL for the tests.

#### 2.3.3. Determination of Minimum Inhibitory Concentrations

The minimum inhibitory concentrations were determined using the broth microdilution method, as described by CLSI in 2012 (protocol M09 A7) coupled with a resazurin-based assay [8]. For this, 6 serial dilutions of each extract and positive control (Amoxicillin) were performed to obtain different concentrations ranging from 2000 to 31.25 **μ**g/mL and from 0.5 to 0.0071 **μ**g/mL, respectively. The final volume in each well was 200 **μ**L and the final concentration of DMSO was less than 1% with no effect on bacteria growth. The test was performed in Muller-Hinton broth (Sigma-Aldrich). The final concentration of the bacterial suspension was 5 × 10^5^ CFU/mL. The negative control was made up with culture media and bacteria suspension, while the sterility control was made up with culture media alone. The plates were covered and incubated at 37°C for 24 hrs. At the end of the incubation period, 20 **μ**L of freshly prepared resazurin (0.15 mg/mL Sigma-Aldrich) was introduced into each well, followed by incubation in the dark for 30 min. At the end of the incubation time, the MIC was defined as the smallest concentration of plant crude extract at which there was no change in coloration from blue to pink, corresponding to the lack of visible bacterial growth.

### 2.4. Phytochemical Screening of Active Extracts

The different groups of secondary metabolites of active extracts were highlighted using colorimetry methods as described by Ciulei in 1982, Wagner and Bladt in 1996, and Hamid et al., in 2018, with minor modifications regarding the solvent of solubilization of the crude extract [9–11]. Tannins and polyphenols were identified by the FeCl_3_ test, flavonoids by the cyanidin reaction, saponosides by the foam test, quinones by the Bornträger test, triterpenes and steroids by the Liebermann–Burchard test, and alkaloids by the Dragendorf test.

### 2.5. Total Phenolics Quantification

Following the procedure outlined by Singleton et al., 1999 [12], the total phenolic contents were determined. In fact, the extracts were dissolved in DMSO at a concentration of 100 mg/mL and then diluted to a final concentration of 1 mg/mL in distilled water. The Folin–Ciocalteu reagent (0.2 N, TECNO PHARMCHEM HARYANA) was added to each test tube corresponding to each dilution that contained 0.5 mL of the solutions obtained after dilution, 2.5 mL of the reagent had been diluted 10 times, and the test tubes were then allowed to sit at room temperature for 5 minutes. Following the incubation time, 2 mL of a sodium carbonate solution (75 mg/mL) was added, and the mixture was then agitated. The test tubes were once again incubated for two hours at room temperature in the dark. The etalon for the calibration curve was made up using gallic at different concentrations of 0, 20, 40, 60, 80, and 100 mg/L. The optical density was obtained by a spectrophotometer (Thermo Scientific Evolution 300) at 760 nm against the blank (0.5 mL of Folin–Ciocalteu reagent and 0.5 mL of sodium carbonate). The test was carried out twice, and the outcome was represented as mg of gallic acid (Sigma-Aldrich) equivalents per 100 mg of extract (mg GAE/100 mg extract) [13].

### 2.6. Total Flavonoid Quantification

The method outlined by Arvouet-Grand et al. in 1994 [14] was used to determine the total flavonoid content. In fact, 2 mL of each extract were mixed with 2 mL of aluminum trichloride (AlCl_3_, Sigma-Aldrich) in methanol (2%), and the mixture was then left to sit at room temperature for 10 min in the dark. The presence of flavonoids was established by the yellow stable coloring that corresponded to the production of a flavonoids-(Al^3+^; 3Cl^−^) complex in comparison to quercetin, which was quantified spectrophotometrically at 415 nm (Thermo Scientific Evolution 300). Based on a linear calibration curve made up with quercetin (Sigma-Aldrich) concentrations ranging from 0 to 100 mg/L, the total flavonoid levels in extracts were determined and expressed in mg quercetin equivalents (mg QE)/100 mg of dry extract [13].

### 2.7. Total Tannin Quantification

According to Ribereau-Gayon and Stonestreet in 1966 and Bate-Smith et al. in 1965 [15, 16], the reaction of Bate-Smith was used to determine the tannin content. Three (3 mL) mL of hydrochloric acid (12 N) were added to 2 mL of each plant's aqueous extract (1 mg/mL) in a hydrolysis tube (glass tube). The tube was then sealed with a Teflon-sealed stopper and heated in a water bath to 100°C for 30 minutes. A control tube with the identical solution inside was kept at room temperature in parallel. After the hydrolyzed tube had cooled, a spectrophotometer was used to measure the optical density at 550 nm (Thermo Scientific Evolution 300). The optical densities were used to calculate the total tannin contents and expressing the results in g/L [13]. The following formula was used to calculate the total tannin content: *C* = 19.33 (Doh-Dot) where *C* is the content of total tannins expressed in g/L, Doh is the optical density of the hydrolyzed tube, and Dot is the optical density of the control tube.

## 3. Results

### 3.1. Plant Research and Extraction Yields

According to traditional medicine, eight herbs from eight families and eight genera are used to treat diarrhea, stomachaches, typhoid fever, gonorrhea, and malaria. The following plant species and their Herbarium number are listed in Table 1. These plants species include *Cassia italica* (Mill.) F. W. Anders, *Limeum pterocarpum* (L.), *Strychnos innocua* Del, *Ipomoea asarifolia* (Desr.), Roem. and Schult, *Blepharis linariifolia* Pers, *Boscia senegalensis* (Pers.) Lam. Ex Poir, *Phyllanthus pentandrus* Schum and Tonn, and *Ximenia americanum* L. The choice of these plants was based on the literature on ethnobotany in Niger, and the results of previous surveys carried out by several researchers were used. Plants used to treat infectious diseases were identified. Those that are more widely used and have not been studied were selected. The different parts (stem bark, leaves, leafy stems, and whole plants) of these plants were harvested for the extract preparation. These plants are widely used in traditional pharmacopeia in Niger. The traditional healer from Niger mainly used water for the remedies' preparation [17–21]. Three solvents were used to prepare 24 extracts, with extraction yields varying from 2.4 to 32.8 percent (Table 2). The extraction yields were dependent on the type of plant, the parts employed, and the extraction solvent. Methanol extraction produced the maximum yield (32.8%) from the stem bark of *X. americana*, followed by water (32.0%) and ethanol (31.2%). In addition, using water as the extraction solvent, the leaves of *Boscia senegalensis* produced a significant yield of 27.5% (Table 2). This finding showed that water and methanol are the best extraction solvents.

### 3.2. Minimum Inhibitory Concentration

The 24 obtained extracts were tested for their antibacterial activity, and the findings are grouped in Table 2. Out of the 24 extracts, 11 (40.74%) from 5 different plant species had antibacterial activity, with MICs varying from 500 *μ*g/mL to 2000 *μ*g/mL. The activity was bacterial and plant extract dependent. The most effective extracts reported came from *P. pentandrus*. With a MIC of 1000 *μ*g/mL, PPME exhibited anti-*Shigella* (*S. flexneri*, *S. sonnei*, and *S. dysenteria*) activity, whilst PPAQ at the same dose reduced the growth of *S. flexneri* and S. sonnei and at 2000 *μ*g/mL inhibited *S. dysenteria*, and PPET at 2000 *μ*g/mL was active against *S. sonnei*. The other plant that inhibited *Shigella* spp. was *X. americana*, and the MIC of most of the extracts from this plant was 2000 *μ*g/mL. According to Table 3, the clinical isolate *S. typhi* was the most susceptible to the investigated extracts, with MICs of 1000 *μ*g/mL (CIME, BLME, and BLET) and 2000 *μ*g/mL (LPME, PPME, and PPET), while PPAQ showed exhibited activity with the MIC of 1000 *μ*g/mL against the clinical isolate of *Salmonella typhimurium*.

### 3.3. Qualitative Phytochemical Screening of Active Extracts

A phytochemical screening was conducted, and the findings are shown in Table 3, to identify the major phytochemical groups in the active extracts that may be accountable for the activity attained. The table suggests that all extracts are high in polyphenols, terpene/sterol, flavonoids, and tannins. Alkaloids are mainly found in *P. pentandrus*. Saponosides were discovered in the methanolic, aqueous, and *X. americana*, *P. pentandrus*, and *B. linariifolia* extracts. Except for the methanolic and ethanolic extracts from *S. innocua*, quinone was present in all extracts (Table 4).

All active extracts contained tannins, flavonoids, and phenolic substances. These substances are referred to as antimicrobial agents. Their quantity was calculated using the regression equation derived from the calibration curves because the activity can occasionally be concentration dependent.

### 3.4. Total Phenolic Compound Content of Plant Extracts

**Figure 1:**
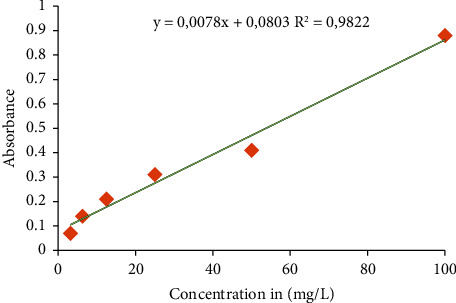
Standard curve of gallic acid.

Gallic acid was used as a standard to obtain the regression equation for the phenolic compound contents, which was *y* = 0.0078*x* + 0.0803 and *R*^2^ = 0.9822 (Figure 1). From this equation, the amount of phenolic compounds in each extract was deducted, and they were then expressed as gallic acid equivalents per 100 mg of dry extract (Table 5). According to Table 5, they range from 8.97 ± 0.09 to 43.59 ± 0.15 mg GAE/100 mg of extract for phenolic components. The highest amount of total phenolic compounds is found in the XAET and XAME extracts, which had 43.59 and 41.97 mg EAG/100 mg, respectively, and were followed by PPET and PPME extracts, which had 29.59 and 27.75 mg EAG/100 mg, respectively.

### 3.5. Total Flavonoid Content of Plant Extracts

**Figure 2:**
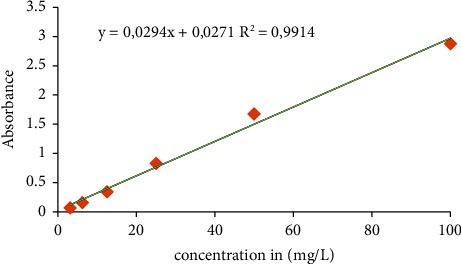
Standard curve of quercetin.

The total flavonoid contents were calculated using the regression curve (Figure 2) obtained using quercetin as a standard and the regression equation *y* = 0.0294*x* + 0.0271 and *R*^2^ = 0.99. The total flavonoid contents were expressed in milligrams of quercetin equivalent per hundred milligrams of dry extract (mg EQ/100 mg). The obtained total flavonoid concentrations ranged from 0.46 EQ/100 mg to 3.12 ± 0.01 EQ/100 mg. The methanolic extract from *P. pentandrus* had the highest total flavonoid concentration, and it was followed by the methanolic extract from *Blepharis linariifolia* (2.970 mg EQ/100 mg).

### 3.6. Total Tannin Content of Plant Extracts

The concentrations of total tannins ranged from 46.49 g/L to 0.36 g/L. With concentrations of 46.49 g/L and 45.52 g/L, respectively, the extracts XAET and XAME displayed the best contents. The total tannin levels of the other extracts were extremely low. The BLET extract showed the lowest content. The extracts that contained the most total tannin did not have the best antibacterial effects. The most effective extracts on the *E. coli* strain are the BLME, PPAQ, and SIME extracts, but they have low quantities of total tannins. These extracts' activity would be related to additional chemical groupings.

## 4. Discussion

Bacteria are the primary cause of diarrhea, which is the second highest cause of death for children under 5 in low- and middle-income nations. The administration of antibiotics is the mainstay of diarrhea therapy. Sadly, the rise of multidrug-resistant bacteria has resulted in treatment failure and keeps raising the mortality toll globally. It is thus vital to carry on looking for fresh substitute agents. Because of this, it has been discovered that some compounds from medicinal plants used in traditional medicine are effective against bacteria pathogens. The goal of the current study was to evaluate the antibacterial properties of several therapeutic plants utilized in Niger's traditional pharmacopeia. According to traditional medicine, eight (8) plants from eight different plant families were employed by traditional healers. Three solvents were used to prepare 24 extracts, which were then tested for antibacterial activity. Of them, 11 showed antibacterial activity with MICs ranging from 2000 to 500 *μ*g/mL. The threshold established by Tamokou et al., 2017 (highly active: MIC ≤100 *μ*g/mL, significantly active: 100 < MIC ≤ 512 *μ*g/mL, moderately active: 512 < MIC ≤ 1024 *μ*g/mL, low activity: 1024 < MIC ≤ 2048 g/mL, and considered not active: MIC > 2048 mg/mL) showed that the activity of extracts ranged from low active to moderately active [22]. These activities vary from one bacterial strain to another and from one extract to another. All extracts tested were inactive against *S. enteridis*, and the aqueous extract of *P. pentandrus* showed moderate activity against *S. typhimurium* with an MIC of 1000 *μ*g/mL. For *E. coli*, the methanolic extracts of *P. pentandrus*, *S. innocua*, and *B. linariifolia* had good activities with MICs ranging from 1000 to 500 *μ*g/mL. Some extracts were significantly active against *E. coli*, including methanolic extracts of *S. innocua* and *B. linariifolia,* which showed the best MIC against this bacterial strain at 500 *μ*g/mL. The methanolic extracts were the most active, and the *P. pentandrus* extracts were the most active. The methanolic extract of *P. pentandrus* was significantly active and showed moderate activity against *Shigella dysenteriae*, *Shigella flexneri*, and *Shigella sonnei*, with an MIC of 1000 *μ*g/mL. The aqueous extract of *P. pentandrus* showed MICs of 1000 *μ*g/mL against *S. flexneri* and *S. sonnei* strains. To our knowledge, the literature does not present data on the anti-*Shigellal* activities of extracts obtained from *P. pentandrus*. The activity obtained could be related to the diversity of phytochemical profiles of extracts having diverse modes of action. Phytochemical screening showed the presence of alkaloids, saponosides, tannins, flavonoids, terpenes/sterols, quinones, and polyphenols. The most active plant was *P. pendandrus,* containing all highlighted secondary metabolite groups except saponosides, followed by *X. americana*, which contained all highlighted secondary metabolite groups except alkaloids. Alkaloids are present only in the extracts of *P. pentandrus*. The samples of the plant *X. americana* showed an absence of alkaloids but the presence of the other groups of secondary metabolites. Alkaloids are known to possess a wide range of biological activities, such as antibacterial, antifungal and antiplasmodial activities. Buphanidrin is an alkaloid that has shown activity against *Bacillus subtilis* (MIC of 13 *μ*g/mL), *S. aureus*, *E. coli*, and *Klebsiella pneumoniae* with an MIC of 63 *μ*g/mL [23]. Quinones are a group of metabolites known to have antimicrobial activities, including antibacterial activities. Quinones isolated from medicinal plants have shown good activities against disease-causing bacteria. Plumbagin isolated from the roots of *D. crassiflora* and *D. canaliculata* (*Ebenaceae*) combined with the efflux pump inhibitor phenylalanine-arginine *β*-naphthylamide (PA*β*N) significantly increased its activity against *E. coli* AG100A with MICs ranging from 2 to 0.5 *μ*g/mL and *E. coli* ATCC 8739 with MICs between 16 and 2 *μ*g/mL [24]. All tested extracts were positive for polyphenols, flavonoids, tannins, and terpenes/sterols, and these different groups of secondary metabolites are important for their various biological activities. Ethanolic and methanolic extracts of *Strychnos innocua* showed the same phytochemical profile but different antibacterial activity profiles. The ethanolic extract of the same plant did not show any activity against the *E. coli* strain, but its methanolic extract showed an MIC of 500 *μ*g/mL, which would be related to the difference in polarity between the two solvents, with methanol being the most polar. These activities would be linked to the presence of other classes of secondary metabolites. Flavonoids, sterols, and terpenes are classes of secondary metabolites known to have antiplasmodial and antibacterial activities; their presence in the extracts of these plants would explain their evaluated biological activities. Tannins have antidiarrheal properties and are enzyme inhibitors, and flavonoids have anti-inflammatory and antioxidant properties [25]. The synergistic action between antidiarrheal and anti-inflammatory properties and inhibition of the development of certain microorganisms is of paramount importance in the fight against certain infectious diseases [25]. The presence of these two groups in the tested extracts should be noted.

This result demonstrated the importance of alkaloid and phenolic compounds in the antibacterial activity. In fact, phenolic compounds act on bacteria by modifying the permeability of cell membranes, the changes in various intracellular functions induced by hydrogen binding of the phenolic compounds to enzymes, or by the modification of the cell wall rigidity with integrity losses due to different interactions with the cell membrane [26]. Alkaloids inhibit bacterial growth through a variety of mechanisms, including inhibition of bacterial nucleic acid and protein synthesis, modification of bacterial cell membrane permeability, damage to the cell membrane and cell wall, inhibition of bacterial metabolism, and inhibition of efflux pumps [27]. Flavonoids are able to link to soluble proteins that are present outside of the cells and with bacterial cell walls, thus promoting the formation of complexes [26, 28]. Flavonoids may also act on bacteria by inhibiting both energy metabolism and DNA synthesis, thus affecting protein and RNA synthesis [29]. The mechanism of action of tannins is explained by their ability to pass through the cell wall up to the internal membrane of the bacteria, their interference with the metabolism of the cell, and as a result, their destruction. It has been shown that tannic acid inhibits bacterial attachment to surfaces [30].

As the antibacterial activity is concentration dependent, the most reported ingredients were quantified using spectrophotometry approaches. Ethanolic and methanolic extracts of *X. americana* contained the most important quantity of phenolic compound (43.59 ± 0.15 and 41.97 ± 0.02 mg EAG/100 mg dry extract, respectively). These same extracts of this plant showed the highest contents of total tannins. For total flavonoids, the highest content was obtained in the methanolic extract of *P. pentandrus* with 3.12 ± 0.01 mg QE/100 mg dry extract, followed by the methanol extract of *B. linariifolia*. The extracts BLET, BLME, SIME, and SIET are the most active against *E. coli*; however, these extracts are not the richest in polyphenols. The activities in these extracts could be related to other classes of compounds or to the diversity of the structures of the phenolic compounds. Low contents of total phenolic compounds were obtained with the SIET and SIME extracts. The extract SIME showed activity against the *E. coli* strain with an MIC of 500 *μ*g/mL. The PPME extract of this plant contained 3.12 mg QE/100 mg, and this extract showed an MIC of 500 µg/mL against the *E. coli* strain. The PPME extract was followed by the BLME and BLET extracts with 2.97 and 2.82 mg EQ/100 mg, respectively; the BLME extract also showed activity against *E. coli* with an MIC of 500 *μ*g/mL. The lowest contents of total flavonoids were observed in the XAET and XAME extracts, with 0.49 and 0.46 mg EQ/100 mg, respectively. The SIME extract shows an activity of 500 *μ*g/mL on the *E. coli* ATCC strain but has a low total flavonoid content of 1.82 mg EQ/100 mg. Flavonoids are a class of secondary metabolites that have key roles in signaling between plants and microbes, in defense as antimicrobial agents and feeding deterrents, and in UV protection [31]. These compounds are found in many plant genera and all parts of plants. They exhibit several biological effects, such as anti-inflammatory, antimicrobial, antiviral, antiulcer, hepatoprotective, antitumor, and antioxidant activities [32]. Many biological properties of flavonoids may be related to their capacity to penetrate into cell membranes and thus affect their biological activity. Isolated flavonoids from *Dorstenia angusticornis* showed good antibacterial activity against some bacterial strains. Bacteriocin C shows good antibacterial activity against *Escherichia coli* with an MIC of 1.22 *μ*g/mL and against *Salmonella typhi* with an MIC of 78.12 *μ*g/mL. Angusticornin B has good antibacterial activity against *Escherichia coli* with MICs of 0.61 *μ*g/mL, 19.53 *μ*g/mL against *Salmonella typhi*, and 0.61 *μ*g/mL against *Shigella dysenteriae*. Stipulin also shows good antibacterial activity against *Shigella dysenteriae*, with MICs of 19.53 *μ*g/mL and 78.12 *μ*g/mL against *Salmonella typhi* [33]. The presence of flavonoids in some extracts could justify the activity of these extracts.

## 5. Conclusion

Five (*Cassia italica*, *Limeum pterocarpum*, *Phyllanthus pentandrus*, *Strychnos innocua*, and *Ximenia Americanum*) of the eight plants showed antibacterial activity, and phytochemical analysis showed the presence of polyphenols, flavonoids, tannins, sterols/terpenes, alkaloids, and quinone in the active extracts. These extracts contain important quantities of total flavonoids, total tannins, and total polyphenols. All these findings could justify the use of these plants in traditional medicine to cure infectious diseases such as diarrhea and demonstrate that these extracts could contain bioactive compounds. Then, further study will be conducted following UHPLC-MS/MS guided isolation to isolate the different unknown compounds responsible for the activity obtained.

## Figures and Tables

**Table 1:** Herbarium number of the eight plants.

Family	Plants species	Herbarium number
*Caesalpiniaceae*	*Cassia italica* (Mill.) F. W. Anders	88
*Molluginaceae*	*Limeum pterocarpum* Lin	1008
*Convolvulaceae*	*Ipomoea asarifolia* (Desr.), Roem. and Schult	242
*Acanthaceae*	*Blepharis linariifolia* Pers	217
*Euphorbiaceae*	*Phyllanthus pentandrus* Schum and Tonn	0347
*Capparidaceae*	*Bossia senegalensis* (Pers) Lam. ex Poir	0432
*Loganiaceae*	*Strychnos innocua* Del	690
*Olacaceae*	*Ximenia americanum* L	28/311

**Table 2:** Medicine plants selected for use in traditional medicine and extraction yields.

Plants	Family	Traditional uses	Part used	Extraction solvent	Codes of extracts	Extraction yields (%) w/w
*Cassia italica*	*Caesalpiniaceae*	Stomach ache, typhoid fevers, urinary, and infections	Whole plant	Methanol	CIME	10.4
				Water	CIAQ	10.0
				Ethanol	CIET	5.8

*Limeum pterocarpum*	*Molluginaceae*	Malaria, bacterial, and fungal infections	Whole plant	Methanol	LPME	9.6
				Water	LPAQ	7.0
				Ethanol	LPET	2.6

*Phyllanthus pentandrus*	*Euphorbiaceae*	Scorpion stings, stomach aches, and infectious diseases	Whole plant	Methanol	PPME	12.8
				Water	PPAQ	24.0
				Ethanol	PPET	8.6

*Strychnos innocua*	*Loganiaceae*	Diarrhea, typhoid fever, and jaundice	Stem bark	Methanol	SIME	8.4
				Water	SIAQ	11.0
				Ethanol	SIET	2.4

*Boscia senegalensis*	*Capparidaceae*	Diarrhea, edema, eczema, and jaundice	Leaves	Methanol	BSME	11.4
				Water	BSAQ	27.5
				Ethanol	BSET	4.8

*Blepharis linariifolia*	*Acanthaceae*	Measles edema, malaria, abscesses, and stomach aches	Whole plant	Methanol	BLME	6.4
				Water	BLAQ	11.0
				Ethanol	BLET	4.0

*Ximenia americanum*	*Olacaceae*	Diarrhea, gonococcal disease, wound, and hemorrhoids	Stem bark	Methanol	XAME	32.8
				Water	XAAQ	32.0
				Ethanol	XAET	31.2

*Ipomoea asarifolia*	*Convolvulaceae*	Typhoid fever, diarrhea, and fungal infections	Whole plant	Methanol	IAME	6.8
				Water	IAAQ	2.5
				Ethanol	IAET	7.2

**Table 3:** Minimum inhibitory concentrations of plant crude extracts.

Minimum inhibitory concentrations (*μ*g/mL)								
Codes extracts	*Bacteria strains*							
	SE CPC	STM CPC	ST CPC	SFNR 518	SONR 519	SD CPC	EC ATCC 25922	EC CPC
CIME	—	—	1000	—	—	—	—	—
CIAQ	—	—	—	—	—	—	—	—
CIET	—	—	—	—	—	—	—	—
LPME	—	—	2000	—	—	—	—	—
LPAQ	—	—	—	—	—	—	—	—
LPET	—	—	—	—	—	—	—	—
PPME	—	—	2000	1000	1000	1000	1000	500
PPAQ	—	1000	—	1000	1000	2000	—	—
PPET	—	—	2000	—	2000	—	—	—
SIME	—	—	—	—	—	—	500	—
SIAQ	—	—	—	—	—	—	—	—
SIET	—	—	—	—	—	—	—	—
BSME	—	—	—	—	—	—	—	—
BSAQ	—	—	—	—	—	—	—	—
BSET	—	—	—	—	—	—	—	—
BLME	—	—	1000	—	—	—	1000	500
BLAQ	—	—	—	—	—	—	—	—
BLET	—	—	1000	—	—	—	—	—
XAME	—	—	1000	—	2000	—	—	—
XAAQ	—	—	—	2000	2000	—	—	—
XAET	—	—	—	—	2000	2000	—	—
IAME	—	—	—	—	—	—	—	—
IAAQ	—	—	—	—	—	—	—	—
IAET	—	—	—	—	—	—	—	—
Amoxicillin	0.976	0.976	1.952	0.976	1.952	0.976	0.976	1.952

SE: *Salmonella enteritidis*, ST: *Salmonella typhi*, STM: *Salmonella typhi*, *Shigella dysenteriae*, *Shigella flexneri*, and *Shigella sonnei*, CPC: *Centre Pasteur du Cameroun*, EC: *Escherichia coli*/ nonactive, CIME: *Cassia italica* methanol, CIET: *Cassia italica* ethanol, CIAQ: *Cassia italica* aqueous, LPME: *Limeum pterocarpum* methanol, LPET: *Limeum pterocarpum* ethanol, LPAQ: *Limeum pterocarpum* aqueous, PPME: *Phyllanthus pentandrus* methanol, PPET: *Phyllanthus pentandrus* ethanol, PPAQ: *Phyllanthus pentandrus* aqueous, SIME: *Strychnos innocua* methanol, SIET: *Strychnos innocua* ethanol, SIAQ: *Strychnos innocua* aqueous, BSME: *Boscia senegalensis* methanol, BSET: *Boscia senegalensis* ethanol, BSAQ: *Boscia senegalensis* aqueous, BLME: *Blepharis linariifolia* methanol, BLET: *Blepharis linariifolia* ethanol, BLAQ: *Blepharis linariifolia* aqueous, XAME: *Ximenia americanum* methanol, XAET: *Ximenia americanum* ethanol, XAAQ: *Ximenia americanum* aqueous, IAME: *Ipomoea asarifolia* methanol, IAET: *Ipomoea asarifolia* ethanol, and IAAQ: *Ipomoea asarifolia* aqueous.

**Table 4:** Results of phytochemical screening of plant extracts.

Code extracts	Alkaloid	Tannins	Flavonoids	Saponosides	Quinone	Terpene/sterol	Polyphenols
PPME	+	+	+	−	+	+	+
PPET	+	+	+	−	+	+	+
PPAQ	+	+	+	+	+	+	+
XAME	−	+	+	+	+	+	+
XAET	−	+	+	+	+	+	+
SIME	−	+	+	−	−	+	+
SIET	−	+	+	−	−	+	+
BLME	−	+	+	+	+	+	+

Absence = −, presence = +, PPME: *Phyllanthus pentandrus* methanol, PPET: *Phyllanthus pentandrus* ethanol, PPAQ: *Phyllanthus pentandrus* aqueous, SIME: *Strychnos innocua* methanol, SIET: *Strychnos innocua* ethanol, BLME: *Blepharis linariifolia* methanol, XAME: *Ximenia americanum* methanol, and XAET: *Ximenia americanum* ethanol.

**Table 5:** Spectrophotometric assay.

Codes extracts	Total polyphenols in mg GAE/100 mg	Total tannins in g/L	Total flavonoids in mg QE/100 mg
PPME	27.75 ± 0.46	3.38 ± 0.14	3.12 ± 0.01
PPET	29.59 ± 0.41	3.12 ± 0.19	2.6 ± 0.07
PPAQ	20.31 ± 0.33	0.79 ± 0.04	2.82 ± 0.09
XAME	41.97 ± 0.02	45.52 ± 0.17	0.46 ± 0.07
XAET	43.59 ± 0.15	46.49 ± 0.53	0.49 ± 0.02
BLET	19.71 ± 0.14	0.36 ± 0.08	2.82 ± 0.01
BLME	20.7 ± 0.08	1.8 ± 0.04	2.97 ± 0.007
SIET	8.97 ± 0.09	1.52 ± 0.05	1.67 ± 0.002
SIME	10.9 ± 0.44	1.27 ± 0.02	1.82 ± 0.07

PPME: *Phyllanthus pentandrus* methanol, PPET: *Phyllanthus pentandrus* ethanol, PPAQ: *Phyllanthus pentandrus* aqueous, SIME: *Strychnos innocua* methanol, SIET: *Strychnos innocua* ethanol, BLME: *Blepharis linariifolia* methanol, BLET: *Blepharis linariifolia* ethanol, XAME: *Ximenia americana* methanol, and XAET: *Ximenia americana* ethanol.

## Data Availability

The data used to support the findings of this study are available from the corresponding author upon request.
